# LINE1 and *Mecp2* methylation of the adult striatum and prefrontal cortex exposed to prenatal immune activation

**DOI:** 10.1016/j.dib.2019.104003

**Published:** 2019-05-23

**Authors:** Paul Basil, Qi Li, Pak-Chung Sham, Grainne M. McAlonan

**Affiliations:** a Department of Psychiatry, The University of Hong Kong, Pokfulam, Hong Kong S.A.R., China; b Department of Molecular & Cellular Biology, Baylor College of Medicine, Houston, TX 77030, USA; c Department of Forensic and Neurodevelopmental Sciences, Institute of Psychiatry, King's College London, De Crespigny Park, Denmark Hill, London SE5 8AF, UK; d Centre for Genomic Sciences, The University of Hong Kong, Pokfulam, Hong Kong S.A.R., China

**Keywords:** Epigenetics, PolyI:C, Methylation, Maternal immune activation, (MIA), Methyl CpG binding protein (*Mecp2)*, Long interspersed Elements-1, (LINE1)

## Abstract

Prenatal exposure to infection and inflammation increases the risk of neurodevelopmental disorders such as schizophrenia and autism. The etiology could be partly through transgenerational and modifiable DNA methylation changes in the adult offspring's brain. This data descriptor presents a dataset of global DNA methylation (using LINE1 assay) and *Mecp2* promoter methylation in adolescent and adult brain tissue of offspring exposed to prenatal immune activation on gestation day 9 and offspring of saline exposed mice. PCR based methylation assays using Sequenom EpiTYPER was used to quantify DNA methylation at promoter CpG methylation of Long Interspersed Elements-1 (LINE1 or L1) and *Mecp2*. The dataset also includes global DNA methylation and *Mecp2* promoter methylation profile at 6 and 12 weeks following early dietary intervention with omega-3 (n-3) PUFA.

**Table:** Specifications table

Subject area	*Neuroscience*
More specific subject area	*Psychiatric epigenetics*
Type of data	*Tables*
How data was acquired	*DNA was isolated and bisulfite converted, Assays were designed to target two genes, data generated using Sequenom EpiTYPER.*
Data format	*Filtered and summarised.*
Experimental factors	*Bisulfite conversion, PCR*
Experimental features	*Genomic DNA was isolated and bisulfite converted. Methylation assays of PCR amplified targets were performed using Sequenom EpiTYPER*
Data source location	*Houston, TX, USA*
Data accessibility	*The described data is included as supplementary material with this article*
Related research article	*Basil P, Li Q, Dempster EL, Mill J, Sham PC, Wong CC* et al. *Prenatal maternal immune activation causes epigenetic differences in adolescent mouse brain. Translational psychiatry 2014; 4: e434.*

**Table:** 

**Value of the data** • This dataset gives the global DNA methylation and Mecp2 profile of adolescent and adult brain exposed to prenatal immune activation. • These epigenetic marks in an animal model relevant to schizophrenia and autism are of importance as they provide mechanistic insights into the impact of environmental risk factors for neurodevelopmental conditions. • Dietary intervention dataset on transposon activity and MECP2 binding in the brain provide preliminary proof of concept that epigenetic effects of neurodevelopmental risk factors may be modifiable.

## 1 Data

**Fig. 1:**
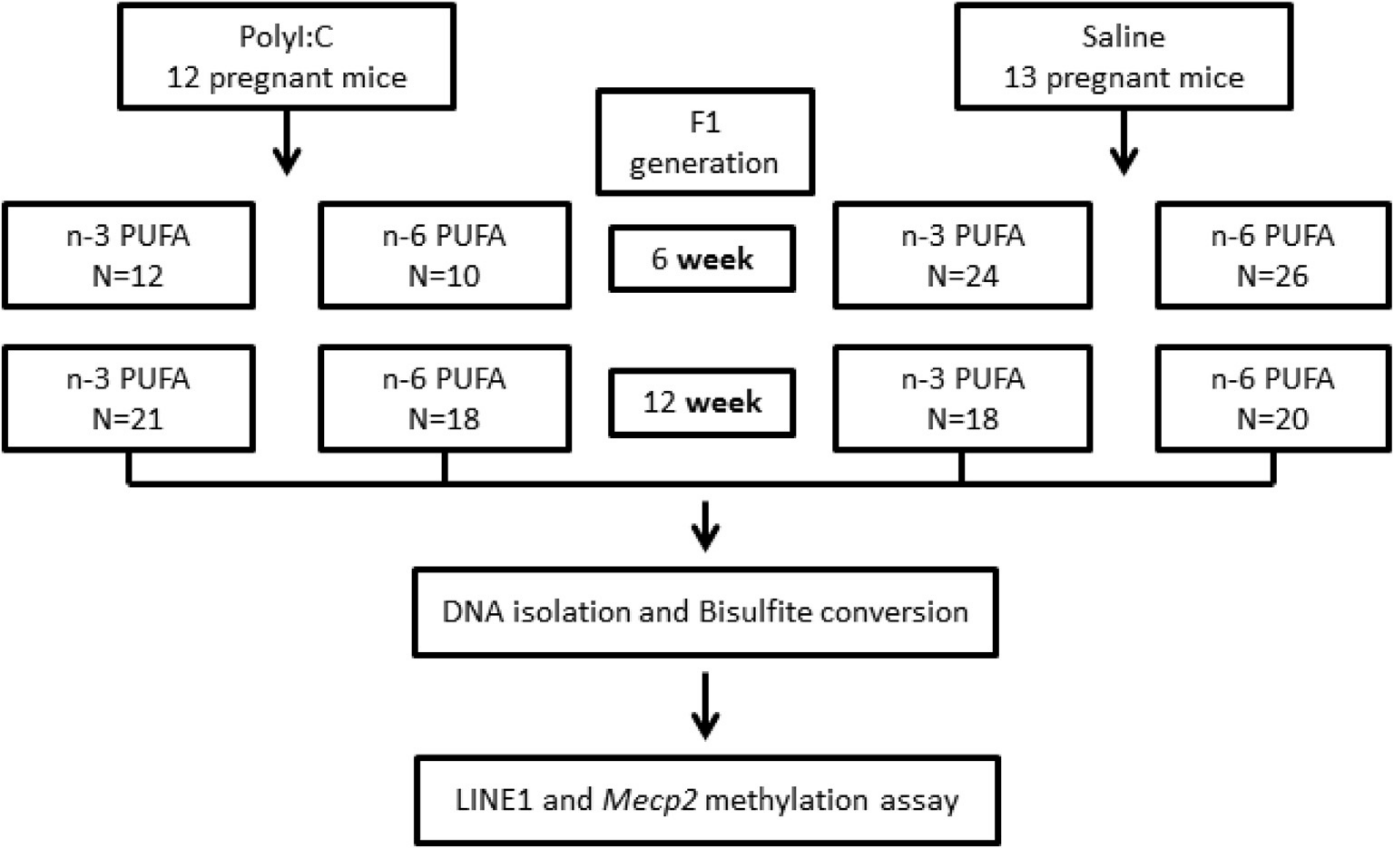
Study design for epigenetic profiling of the MIA mouse model following dietary intervention.

This manuscript describes methylation datasets from offspring exposed to prenatal infection and subsequent inflammation in the striatum and prefrontal cortex (prenatal inflammation group: n = 61; males = 34, females = 27) and matched controls (control group: n = 88; males = 39, females = 49). Half of the animals in each group received dietary intervention with n-3 poly unsaturated fatty acids (PUFA) from weaning. Fig. 1 shows the study design and Table 2 lists the composition of the diets used. Methylation data was generated from 6-week (equivalent to adolescent) and 12-week (equivalent to adult) mouse brain tissues of interest. Genomic DNA was extracted from the samples listed in Table 1 using Qiagen EZ1 DNA extraction protocol and bisulfite converted. LINE1 and *Mecp2* target regions were amplified by PCR using primers listed in Table 3. CpG sites in these amplicons were assayed using Sequenom EpiTYPER platform. Mean LINE1 and *Mecp2* promoter methylation data in PFC and striatum are listed in supplementary tables. Lists are sorted on ID column with summary information (brain region, age, diet and group). Other columns are body weight in grams at 12 week or 6 week, MIA group or saline control group, assigned diet (n-3 or n-6), sex, mean LINE1 promoter methylation, mean *Mecp2* promoter methylation. Missing data marked as ‘na’ are either not measured or samples with <70% data.

**Table 2:** Table of contents of different diet compositions used.

Contents (%)	n-3	n-6	AIN-93
Protein	18.30	18.30	20
Fat	7.10	7.10	5.6
LA	1.20	3.72	2.19
n-3	3.5	0.5	0.33
PUFA	3.08	3.92	3.78
n-3:n-6	1:1	0.08:1	0.09:1

**Table 1:** Number of animals used in this dataset.

	Saline		PolyI:C	
	Male	Female	Male	Female
6-week	20	30	16	6
12-week	19	19	18	21

**Table 3:** Primers used in different assays.

Assay	Forward Primer with *Tag - AGGAAGAGAG*	Reverse Primer with *Tag - CAGTAATACGACTCACTATAGGGAGAAGGCT*	Annealing Temp (ºC)	Amplicon Length (bps)	CpGs Covered
LINE-1	GATTTTAAGATTTTTGGTGAGTGGA	AAAAACTTATACCCCAAATCAAACC	55	119	5
*Mecp2*	GATTAGTTTGTGTGTTGTTGTATTTG	AAAAACCCAATTAATCCTCAACATT	55	282	11

## 2 Experimental design, materials and methods

C57BL/6 N mice were bred and mated in the Laboratory Animal Unit (LAU), The University of Hong Kong. The animals were maintained under ad libitum food and water, kept in 12:12 h normal light-dark cycle (lights off at 19:00) and temperature and humidity-controlled (21 ± 1 °C, 55 ± 5%) animal vivarium. Pregnant females were not disturbed, except for weekly cage cleaning. All experiments were performed in accordance with relevant institutional and national guidelines and regulations approved by the Committee on the Use of Live Animals in Teaching and Research (CULATR) at The University of Hong Kong and every effort was made to minimize the number of animals used and their suffering.

PolyI:C administered C57BL6N MIA mouse model was generated as described elsewhere [1], [2]. In short, a sodium salt of polyI:C was administered on gestation day-9 (GD9) via the tail vein under mild physical constraint [3]. The animals were returned to the home cage after injection and were not disturbed until postnatal day (PND) 21.

### 2.1 Experimental animals and PUFA administration

The pups were weaned, weighed, and littermates of the same sex were caged three to four per cage on PND 21. Both saline control group and polyI:C group were split into two halves with diet enriched with n-3 was matched to a standard rodent American Institute of Nutrition 93 (AIN93) diet or diet enriched with n-6. Diets differ only in the ratio of n-3/n-6 fatty acids used and provides 16% energy from fat diets supplied by Harlan Laboratories, Madison, and water. Please see study design Fig. 1. Description of the total number of animals used is shown in Table 1.

Approximately 2 gm n-3/day was administered through diet as an early dietary intervention. The calorific value and total fat content of both n-3 and n-6 diet was balanced. A detailed list of nutrient contents of the rodent AIN93 and modified diet is shown in Table 2.

### 2.2 Dissection, tissue collection and DNA extraction

At 6-week and 12-week of age, the mice were sacrificed by cervical dislocation and brains removed quickly and transferred to chilled PBS solution. Striatum and prefrontal cortex were collected in 1.5ml tubes using microdissection on a cold platform referring to the Allen Mouse Brain Atlas [4] and flash frozen in liquid nitrogen for storage. DNA was extracted in EZ1 Advanced XL using Qiagen EZ1 DNA extraction kit as per manufacturer's protocol. All DNA samples underwent quantification using Nanodrop spectrometry and quality control assessment using gel electrophoresis before being used in the bisulfite conversion.

### 2.3 Bisulfite conversion

EZ DNA methylation kit from Zymo Research, CA, USA was used to treat five hundred nanograms of genomic DNA with sodium bisulfite in duplicate following the manufacturer's standard protocol. The kit exploits the three-step chemical modification that converts unmethylated cytosine to uracil and the methylated cytosine will be protected from sodium bisulfite [5].

### 2.4 PCR based Global and Candidate gene Methylation Assays

LINE1 elements and *Mecp2* promoter were amplified using previously reported primers in Table 3 from Sigma-Aldrich, UK for analysis with the Sequenome EpiTYPER, CA, USA. PCR products in duplicates were pooled together for reducing PCR bias and EpiTYPER assay was performed. Quantitative DNA methylation was measured using LINE1 assay that gives a proxy of global methylation across ∼600,000 repeats in the mouse genome [6] including + ve and –ve controls for all assays. Assays were carried out on a Sequenome EpiTYPER platform [7] using universal methylated DNA as a methylated reference (EMD Millipore Corporation), and an unmethylated DNA as negative control. The LINE1 assay was designed to cover a consensus sequence. However, due to possible variations in the genomic sequences in these locations, the assays may not cover all instances across the genome.

### 2.5 EpiTYPER data analysis

MALDI-TOF MS readings were interpreted by EpiTYPER software and generates quantitative information about individual CpGs in each analyzed amplicon. Blank, fully methylated and fully unmethylated controls were confirmed for their corresponding epigram and methylation levels. CpGs with missing data (>20%) and samples with less than 70% data recorded across the CPGs were deemed unfit for subsequent analysis. All flagged data from EpiTYPER such as low mass, high mass (outside MS analytical window) were discarded. Mean CpG methylation for LINE1 and *Mecp2* is provided in the dataset as values from 0 to 1 (0 represents not methylated and 1 represent fully methylated).
