# Supplementary material for: LINE1 and Mecp2 methylation of the adult striatum and prefrontal cortex exposed to prenatal immune activation
Source: Data Brief. 2019 May 23;25:104003. doi: 10.1016/j.dib.2019.104003 (PMC6545381; doi:10.1016/j.dib.2019.104003)
Supplement: Supplementary file 1 — Multimedia component 1 [file mmc1.pdf]

03/25/2019

To: Dr. Hao-Ran Wang and Dr. Ganhui Lan

Editors-in-Cheif,

Data in Brief

Re: **LINE1 and *Mecp2* methylation of the adult striatum and prefrontal cortex exposed to prenatal immune activation.** Basil et al.

Dear Drs. Hao-Ran Wang and Ganhui Lan,

On behalf of all the authors I wish to confirm that there are no known conflicts of interest associated with this publication and there has been no significant financial support for this work that could have influenced its outcome.

With many thanks for your time and consideration.

Yours sincerely,

Paul Basil B.Sc. M.Sc. PhD

Molecular and Cellular Biology, Baylor College of Medicine, One Baylor Plaza, Houston, TX, 77030 USA.

Email: basil.paul@bcm.edu

Tel: +1 713-798-6232

Fax: +1 713-790-1275
